# Supplementary material for: Arabidopsis late blight: infection of a nonhost plant by Albugo laibachii enables full colonization by Phytophthora infestans
Source: Cell Microbiol. 2016 Jul 8;19(1):e12628. doi: 10.1111/cmi.12628 (PMC5215655; doi:10.1111/cmi.12628)
Supplement: Supplementary file 1 — Supporting info item [file CMI-19-0-s001.zip › Belhaj_et_al_supplements_figs_tables_revised.pdf]

Fig. S1

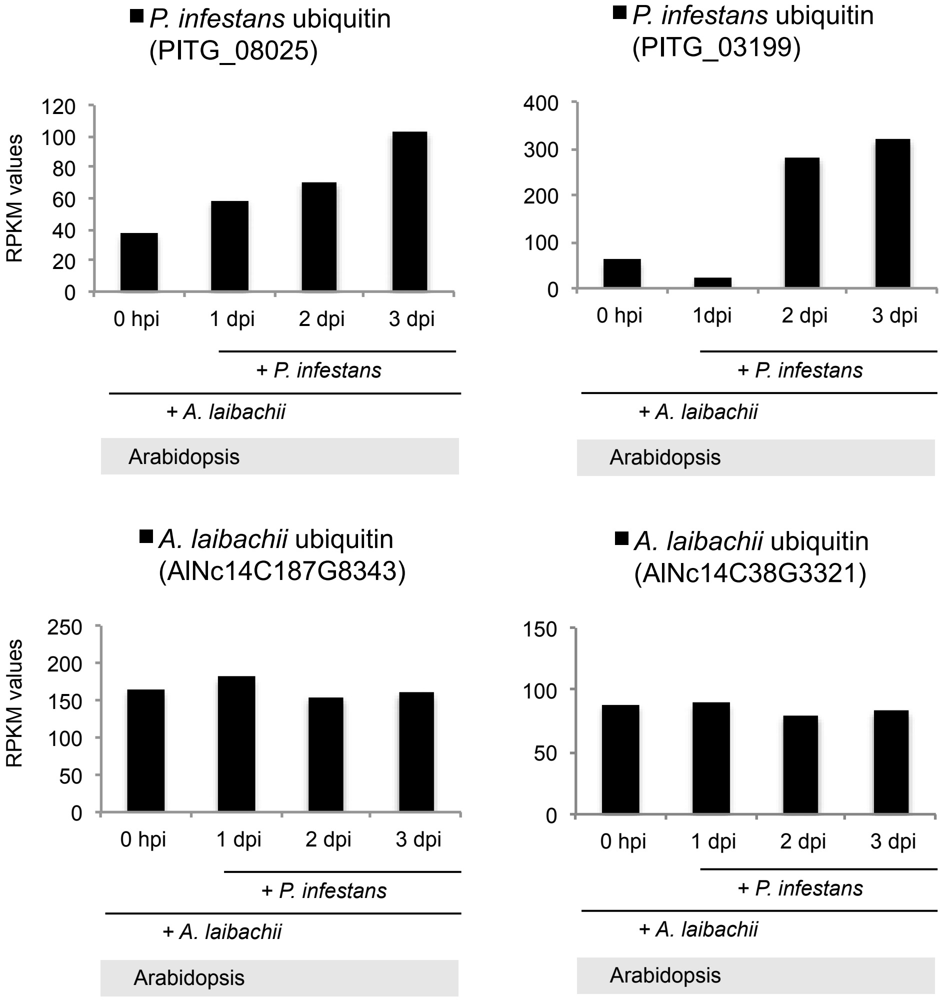

Supplementary Figure 1. **Gene expression as reads per kilobase per million mapped reads (RPKM) values for two ubiquitin control genes in *P. infestans* strain 3928A and *Albugo laibachii* strain NC14 during co-infection in *A. thaliana* Col-0.** RPKM values were obtained from RNAseq reads counts using HTSeq program. RPKM values show that the expression of two *P. infestans* ubiquitin control genes PITG_08025 and PITG_03199 increases over time but the expression of two other similar control genes in *A. laibachii* AlNc14C187G8343 and AlNc14C38G3321 are maintained. The expression of these ubiquitin genes was used as markers to measure accumulation of biomass of *Phytophthora infestans* and *Albugo laibachii* during co-infection on *A. thaliana*.

Fig. S2

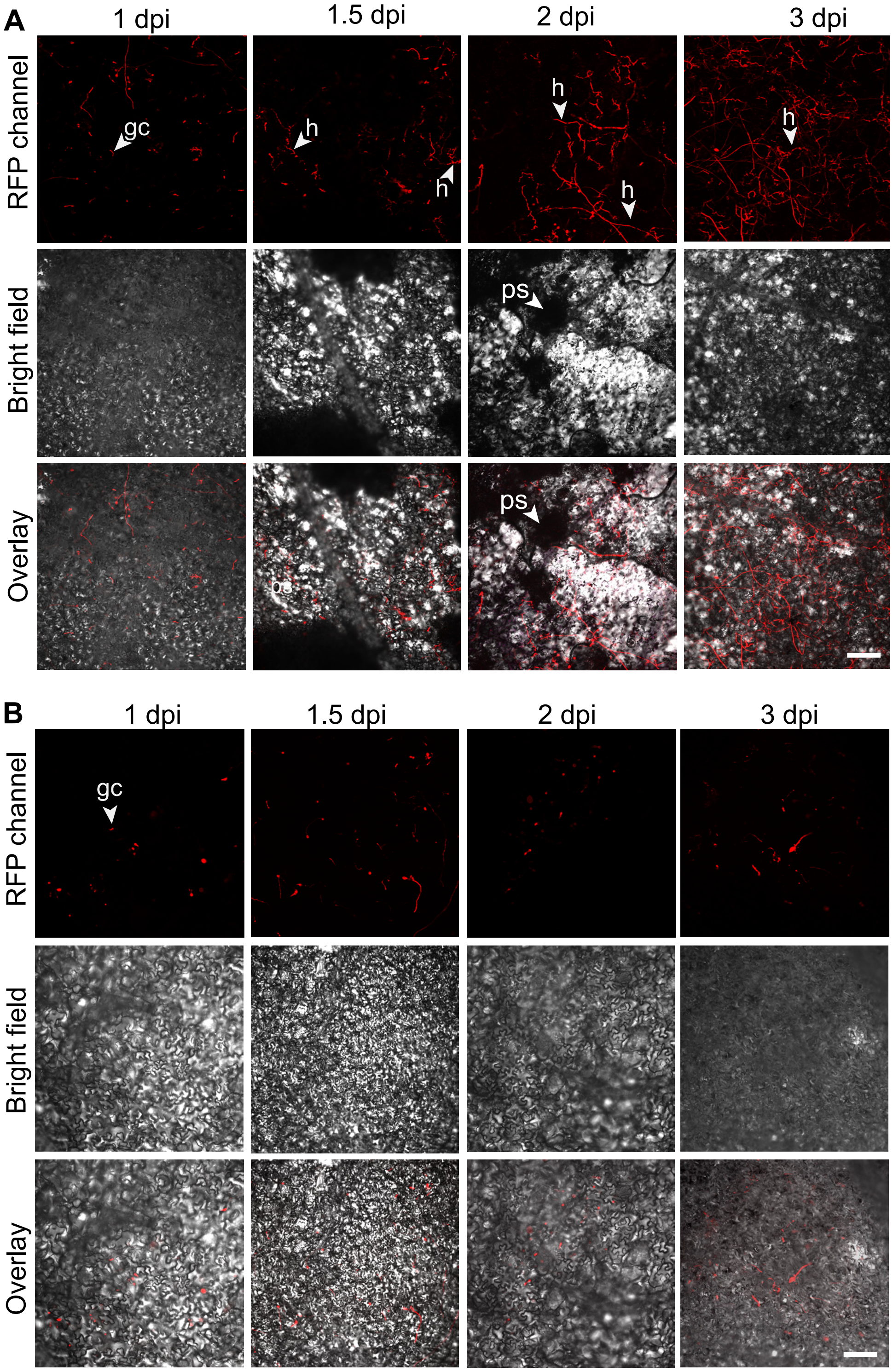

Supplementary Figure 2. *A. laibachii* pre-colonization supports formation of *P. infestans* infection structures in Arabidopsis beyond the surface penetration stage. Time course of red fluorescent *P. infestans* 88069td infection on *A. thaliana* Col-0 pre-infected with *A. laibachii* or mock-treated with water. **(A)** *A. laibachii* pre-colonized *A. thaliana* shows the first hyphae of *P. infestans* 88069td inside the leaf at 1.5 dpi and extensive intercellular colonization of *A. thaliana* Col-0 leaf mesophyll at 2 and 3 dpi. **(B)** *P. infestans* does not grow on *A. thaliana* control leaves. Experiment was performed twice with similar results. Scale bars = 250 μm (A, C) or 50 μm (B). Abbreviations: h: hyphae of *P. infestans*; gc: germinating cyst of *P. infestans;* ps: pustules of *A. laibachii*.

Fig. S3.

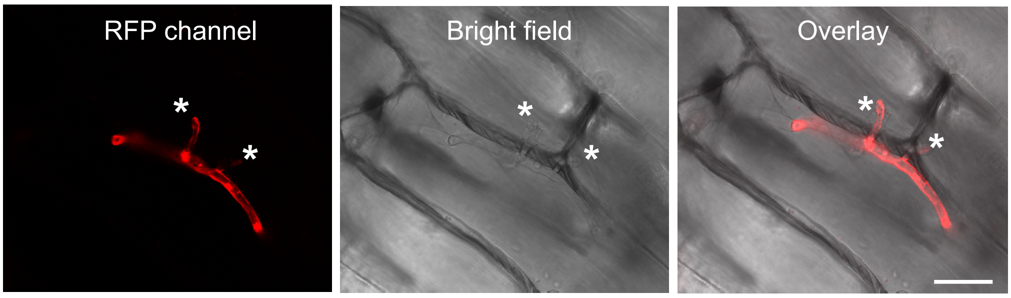

Supplementary Figure 3. *P. infestans* can form haustoria in the nonhost plant Arabidopsis pre-treated with *A. laibachii.* *A. thaliana* Col-0 precolonized with *A. laibachii* was inoculated with red fluorescent *P. infestans* 88069td. Inspection by microscopy at 2 dpi revealed the presence of digit-like haustoria of *P. infestans* independently of *A. laibachii*. Experiment was performed twice with similar results. Abbreviations: #: haustoria of *A. laibachii*, *: haustoria of *P. infestans.* Scale bar = 10 μm.

Fig. S4.

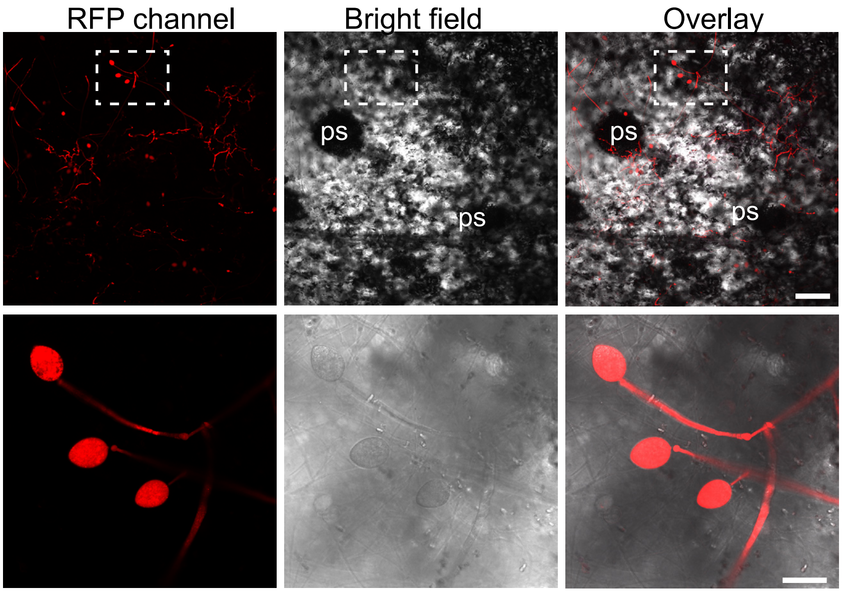

Supplementary Figure 4. *P. infestans* can extensively colonize and sporulate on nonhost Arabidopsis precolonized with *A. laibachii*. *A. thaliana* Col-0 precolonized with *A. laibachii* was inoculated with red fluorescent *P. infestans* 88069td and imaged at 3 days post inoculation with confocal laser scanning microscopy. The upper panel shows confocal micrographs of hyphal extension and sporulation of *P. infestans in A. laibachii* pre-treated Arabidopsis leaves. The lower panel is a closeup of the region highlighted by the dotted square in the upper panel and shows *P. infestans* emerging sporangiophores from the leaf surface, giving rise to lemon-shaped zoosporangia. Experiment was performed twice with similar results.
Scale bar = 100 μm (upper panel) or 10 μm (lower panel).

Fig. S5.

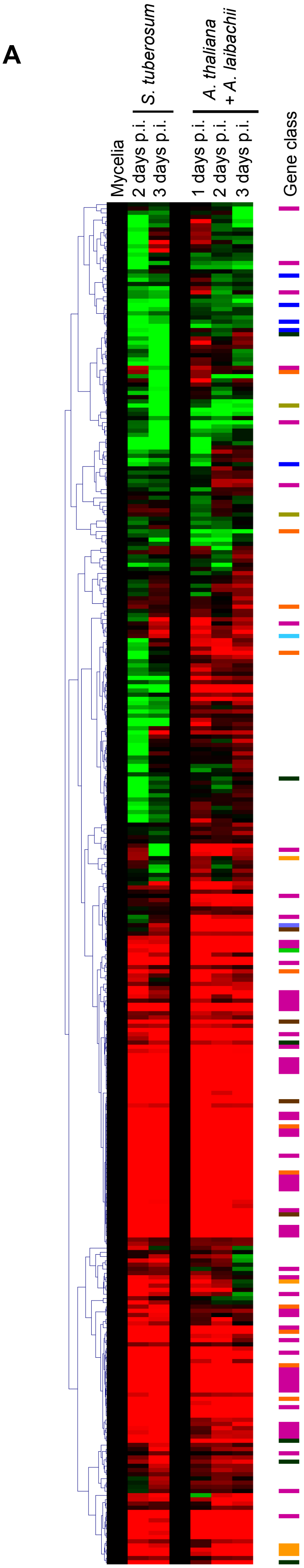

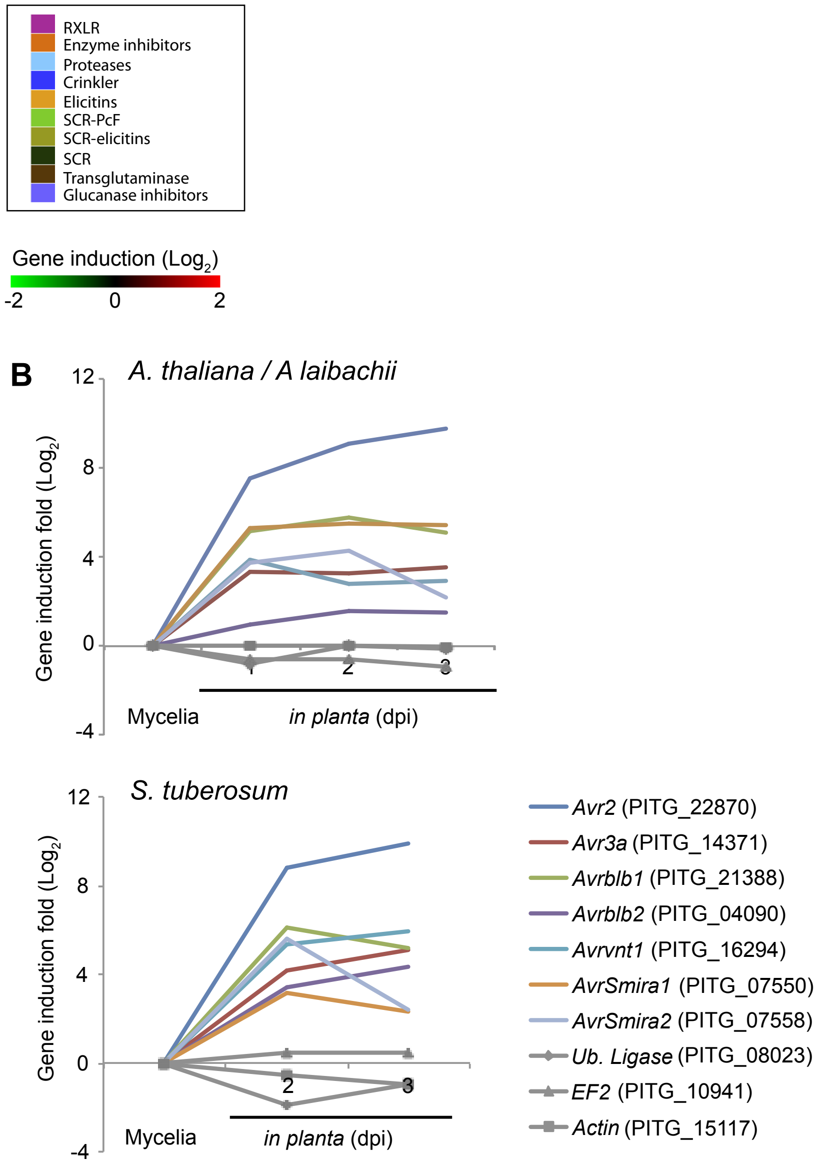

Supplementary Figure 5. *P. infestans* genes encoding secreted proteins during infection of potato and Arabidopsis pre-colonized with *A. laibachii*.

The heat map **(A)** illustrates 325 genes encoding secreted proteins; these genes were induced at least 2-fold during the interaction of *P. infestans* with *S. tuberosum* leaves at 2 dpi and 3 dpi and during the interaction with Arabidopsis leaves colonized with *A. laibachii* at 1, 2, and 3 dpi. These genes were mean-centered and hierarchically clustered by Euclidean distance. **(B)** Expression dynamics of selected *P. infestans AVR* genes and constitutively expressed control genes at 1, 2, and 3 days post inoculations (dpi).

Fig. S6.

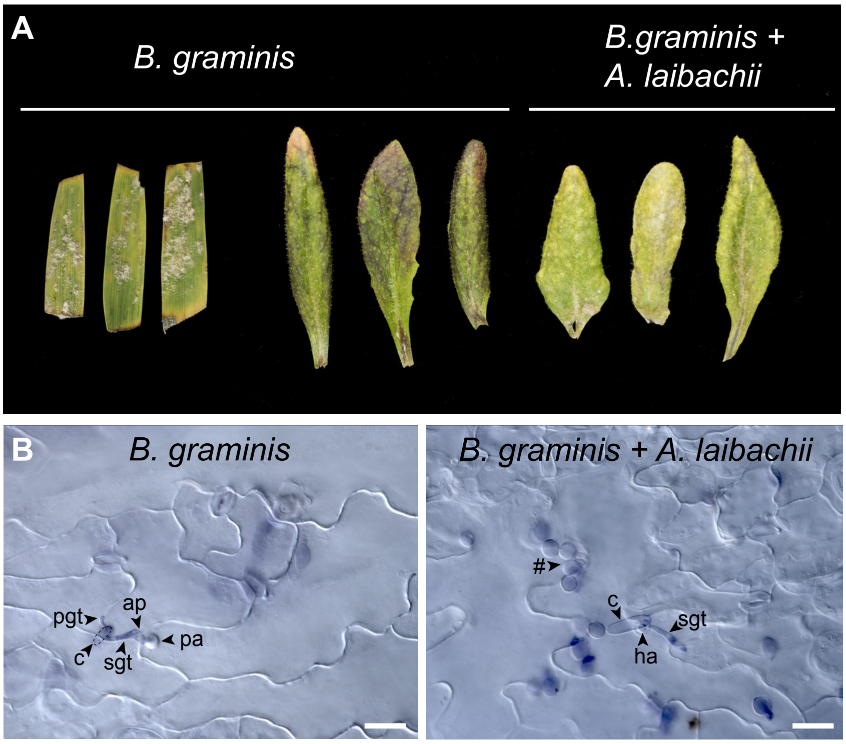

Supplementary Figure 6. *A. laibachii* does not enable the nonhost powdery mildew pathogen to infect nonhost Arabidopsis. *A. thaliana* Col-0 mock-treated (control) or precolonized with *A. laibachii* was inoculated with *Blumeria graminis* f.sp *hordei* (Bgh) isolate CH4.8. The susceptible Barley cv. Golden Promise was used as a control for the infection. **(A)** Macroscopic phenotype of disease symptoms two weeks post inoculation with Bgh isolate CH4.8. **(B)** Maximum projection of images produced by light microscopy from 14 Z-stacks from infected tissues. Micrographs show fungal structures 2 weeks post infection in both control (right panel) and samples pre-colonized with *A. laibachii* (left panel) and reveal that the fungus was stopped at the penetration stage in both interactions. Experiment was performed twice with similar results. Abbreviations: c: conidium, pgt: primary germ tube, sgt: secondary germ tube, ap:appressorium, pa: papillae, ha: haustoria of *A. laibachii,* #: conidiospores of *A. laibachii.* Scale bar = 10 μm.

Fig. S7.

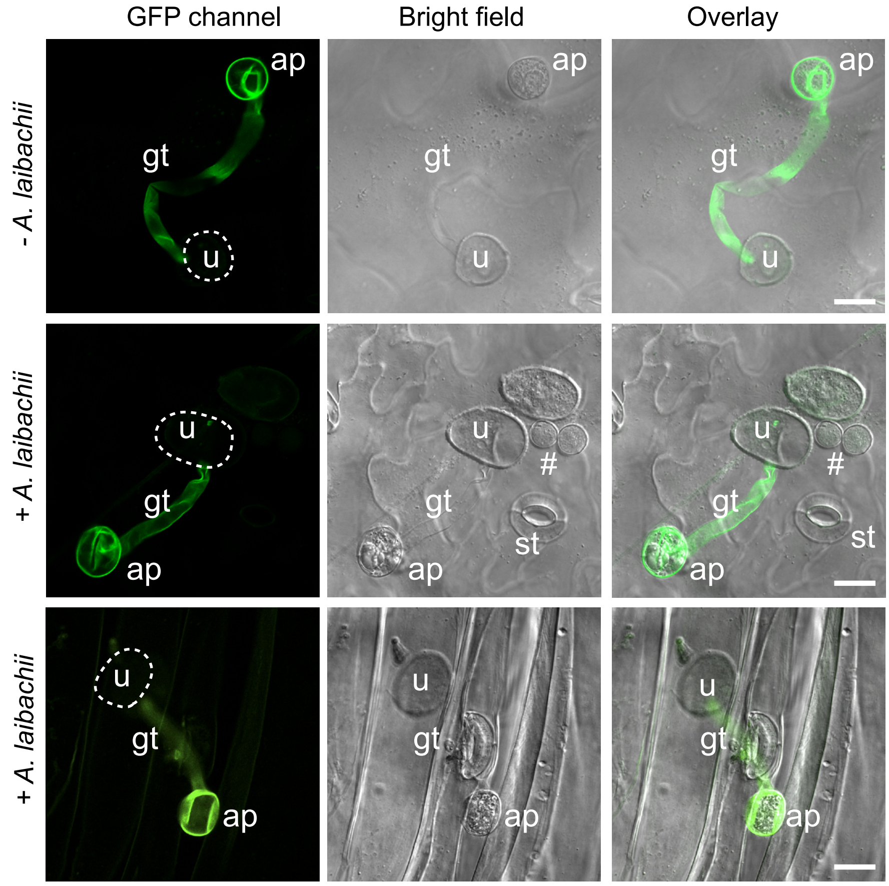

Supplementary Figure 7. *A. laibachii* does not enable the nonhost Asian soybean rust to infect nonhost Arabidopsis Five-week-old *A. thaliana* Col-0 mock-treated (control) or precolonized with *A. laibachii* was inoculated with spore suspension of *Phakopsora pachyrhizi* isolate PPUFV02 and incubated in high humidity. Pathogen structures were visualized with confocal laser scanning microscopy under GFP illumination at 6 days post inoculation. The micrographs show uredisniospores germinating on the leaf surface, producing an appressorium with no further growth in both control (upper panel) and pre-colonized samples with *A. laibachii* (middle and lower panels). All experiments were performed twice with similar results.

Abbreviations: u: urediniospore, ap:appressorium, st: stomata, #: conidiospores of *A. laibachii.* Scale bar = 10 μm.

Supplementary Table 1. List of expressed genes in both potato/*P.infestans* and *A. thaliana*/*A. laibachii*-*P. infestans* interactions. The file compiles three different worksheets containing lists (list 1-3) of genes of *P. infestans* and their expression profiles during infection in potato (List 1) and/or in *A. thaliana* preinfected with *A. laibachii* (List 2-3). List 1 comprises 10,698 coding genes of *P. infestans* on potato. List 2 comprises 7118 coding genes that are expressed in *A. thaliana* preinfected with *A. laibachii* only when compared to potato - *P. infestans* interaction. List 3 is a set of 325 *in planta*-induced genes encoding secreted proteins in both treatments (potato and pre-infected *A. thaliana* with *A. laibachii*).

Abbreviations: GSR: gene sparse region, GDR: gene dense region, InBW: in between in between gene sparse and gene dense, NA: not applicable. If an NA is present in a column with annotations, this refers to no annotation to that type or description. If NA is present in a column with expression data this refers to a lower expression than 2-fold (or log2 <1) and in consequence it was not considered as *in planta* induced for that particular gene.

Supplementary Table 2. Pathogen isolates and media or susceptible plants used for maintaining pathogens

Pathogen species	Isolate name	Maintenance on	Reference
Abugo laibachii	NC14	Arabidopsis Col-gl RPW8.1 RPW8.2	(Thines *et al.*, 2009)
Phytophthora infestans	88069td	RSA media + antibiotics	(Whisson *et al.*, 2007)
P. infestans	06_3928A	RSA media	(Cooke *et al.*, 2012)
Blumeria graminis f.sp. hordei	CH4.8 (IPKBgh)	Barley *cv.* Golden Promise	(Brown *et al.*, 1990)
Phakopsora pachyrhizi	PPUFV02	Soybean *cv.* Ankur (accession PI462312)	(Langenbach *et al.*, 2013)

*Abbreviations: RSA, rye sugar agar; *cv.,* cultivar.

References

Brown, J. and Wolfe, M. (1990). Structure and evolution of a population of Erysiphe graminis f. sp. hordei. *Plant Pathology* **39,** 376-390.

Cooke, D.E., Cano, L.M., Raffaele, S., Bain, R.A., Cooke, L.R., Etherington, G.J., *et al.* (2012). Genome analyses of an aggressive and invasive lineage of the Irish potato famine pathogen. *PLoS Pathog* **8,** e1002940.

Langenbach, C., Campe, R., Schaffrath, U., Goellner, K. and Conrath, U. (2013). UDP-glucosyltransferase UGT84A2/BRT1 is required for Arabidopsis nonhost resistance to the Asian soybean rust pathogen Phakopsora pachyrhizi. *New Phytologist* **198,** 536-545.

Thines, M., Choi, Y.J., Kemen, E., Ploch, S., Holub, E.B., Shin, H.D. and Jones, J.D. (2009). A new species of Albugo parasitic to Arabidopsis thaliana reveals new evolutionary patterns in white blister rusts (Albuginaceae). *Persoonia* **22,** 123-128.

Whisson, S.C., Boevink, P.C., Moleleki, L., Avrova, A.O., Morales, J.G., Gilroy, E.M., *et al.* (2007). A translocation signal for delivery of oomycete effector proteins into host plant cells. *Nature* **450,** 115-118.

Supplementary Table 3: Alignment statistics of RNAseq reads obtained from the dual interaction of *P. infestans*-*S. tuberosum* and the tripartite interaction of *A. laibachii*-*P. infestans*-*A. thaliana*. Illumina paired-end reads of 76bp length were aligned to the reference genome assembly of *P. infestans* strain T30-4 using TopHat software.

Run ID	Lane ID	Sample name*	Total No. of reads** (76bp X 2)	No. of mapped reads	% of mapped reads	No. of unmapped reads	% of unmapped reads
ID146	Lane 3	Pinf_myRSA	71,232,060	69,542,961	97.63	1,689,099	2.37
ID124	Lane 7	Pinf_Stub_2dpi	75,790,960	1,857,167	2.45	73,933,793	97.55
ID124	Lane 8	Pinf_Stub_3dpi	72,939,282	7,602,390	10.42	65,336,892	89.58
ID155	Lane 2	Alai_Atha_Pinf_1dpi	82,411,680	202,205	0.25	82,209,475	99.75
ID155	Lane 3	Alai_Atha_Pinf_2dpi	77,921,536	409,474	0.53	77,512,062	99.47
ID155	Lane 4	Alai_Atha_Pinf_3dpi	57,795,332	297,330	0.51	57,498,002	99.49

*Pathogen and plant species sample abbreviations. Alai: *Albugo laibachii* isolate NC14, *Phytophthora infestans* genotype 13_A2 isolate 06_3928A, Atha: *Arabidopsis thaliana* ecotype Col0, *Solanum tuberosum* cultivar Desiree.

**Number of RNAseq reads after quality control. Reads without Ns and without abnormal length (other than 76bp).
